# CRISPR/Cas9 as a therapeutic tool for triple negative breast cancer: from bench to clinics

**DOI:** 10.3389/fmolb.2023.1214489

**Published:** 2023-07-04

**Authors:** Prashant Kumar Tiwari, Tin-Hsien Ko, Rajni Dubey, Mandeep Chouhan, Lung-Wen Tsai, Himanshu Narayan Singh, Kundan Kumar Chaubey, Deen Dayal, Chih-Wei Chiang, Sanjay Kumar

**Affiliations:** ^1^ Biological and Bio-Computational Lab, Department of Life Sciences, Sharda School of Basic Science and Research, Sharda University, Greater Noida, Uttar Pradesh, India; ^2^ Department of Orthopedics, Taipei Medical University Hospital, Taipei City, Taiwan; ^3^ Division of Cardiology, Department of Internal Medicine, Taipei Medical University Hospital, Taipei City, Taiwan; ^4^ Department of Medicine Research, Taipei Medical University Hospital, Taipei City, Taiwan; ^5^ Department of Information Technology Office, Taipei Medical University Hospital, Taipei City, Taiwan; ^6^ Graduate Institute of Data Science, College of Management, Taipei Medical University, Taipei City, Taiwan; ^7^ Department of Systems Biology, Columbia University Irving Medical Centre, New York, NY, United States; ^8^ Division of Research and Innovation, School of Applied and Life Sciences, Uttaranchal University, Dehradun, Uttarakhand, India; ^9^ Department of Biotechnology, GLA University, Mathura, Uttar Pradesh, India; ^10^ Department of Orthopedic Surgery, School of Medicine, College of Medicine, Taipei Medical University, Taipei City, Taiwan

**Keywords:** triple negative breast cancer, CRISPR/Cas9, gene editing, immunotherapy, drug resistance and artificial intelligence

## Abstract

Clustered regularly interspaced short palindromic repeats (CRISPR) is a third-generation genome editing method that has revolutionized the world with its high throughput results. It has been used in the treatment of various biological diseases and infections. Various bacteria and other prokaryotes such as archaea also have CRISPR/Cas9 systems to guard themselves against bacteriophage. Reportedly, CRISPR/Cas9-based strategy may inhibit the growth and development of triple-negative breast cancer (TNBC) via targeting the potentially altered resistance genes, transcription, and epigenetic regulation. These therapeutic activities could help with the complex issues such as drug resistance which is observed even in TNBC. Currently, various methods have been utilized for the delivery of CRISPR/Cas9 into the targeted cell such as physical (microinjection, electroporation, and hydrodynamic mode), viral (adeno-associated virus and lentivirus), and non-viral (liposomes and lipid nano-particles). Although different models have been developed to investigate the molecular causes of TNBC, but the lack of sensitive and targeted delivery methods for *in-vivo* genome editing tools limits their clinical application. Therefore, based on the available evidences, this review comprehensively highlighted the advancement, challenges limitations, and prospects of CRISPR/Cas9 for the treatment of TNBC. We also underscored how integrating artificial intelligence and machine learning could improve CRISPR/Cas9 strategies in TNBC therapy.

## 1 Introduction

Cancer is characterized by uncontrolled cell division, a failure of cell cycle checkpoints, and mutations in the tumor suppressor gene (TSG) (Matthews et al., 2022). Of various cancer types, breast cancer is the most frequent type of cancer in women, with a higher mortality rate worldwide (Waks and Winer, 2019). Breast cancer is a heterogeneous disease with several distinct entities such histological and biological traits, clinical manifestations and behaviors, and therapeutic responses (Weigelt et al., 2010). The classification of breast cancer provides an accurate idea for the diagnosis and tumor prediction. The use of common biomarkers and clinicopathologic characteristics has been the main criteria to classify breast cancer (Tsang and Tse, 2019). The breast cancer prognosis and treatment response are affected by a variety of factors, including the presence of the estrogen receptor (ER), the progesterone receptor (PR), human epidermal growth factor receptor 2 (HER2/neu), tumor histological grade, type, and size, and the lymph node metastasis (Al-Thoubaity, 2020). There are five identified intrinsic breast cancer molecular subtypes, including luminal A, luminal B, HER2-enriched, Basal-like, and claudin-low (Prat et al., 2015). TNBC is molecular subtype of cancer where all ER, PR, and HER2 remain unexpressed (Yin et al., 2020). These pathological characteristics are associated with TNBC, supporting its rapid progression, and more aggressive behavior than any other type of breast cancer (Feng et al., 2018). Additionally, Perou et al. (2000) used microarray technology to reclassify breast cancer and identify five intrinsic subtypes of breast cancer (Cadenas, 2012). Basal-like breast cancer is the subtype of breast cancer that behaves as triple-negative phenotype, and associated with rapid progression. Notably, all basal-like breast cancer is usually misinterpreted as TNBC; however, only 77% of them are TNBC. On the contrary, 71%–91% TNBCs are basal-like, indicating that both types of breast cancer overlap and present distinct classifications (Wang D.-Y. et al., 2019). This necessitates to characterize the TNBC heterogeneity to clarify prognosis and identify potential responders to current and future treatments. Also, TNBC represents 15%–20% of total breast cancer, which is more common in women younger than age 50. BRCA1 or BRCA2 mutations have been reported in approximately 20% of TNBC (Xie et al., 2017; Tzikas et al., 2020). It has been also shown that TNBC exhibit a special immune microenvironment that includes high levels of vascular endothelial growth factors, tumor-associated macrophages (TAMs), tumor-infiltrating lymphocytes (TILs), and other molecules that are involved in tumour growth and migration. Hence, it is crucial to comprehend the TNBC microenvironment for its prognosis and treatment (Fan and He, 2022).

To evaluate the prognosis and ensure effective treatment for TNBC, an accurate diagnosis is highly important which is mainly based on immunohistochemistry (IHC) to detect ER, PR, and HER2, in addition to mammography to find out the lumps in the breast. However, mammography can not sufficiently produce the image of intra-tumoral characteristics, such as necrosis and fibrosis (Deepak Singh et al., 2021). Numerous strategies are being used to upgrade TNBC patient care in, owing to poor prognosis and diagnosis, which limits clinicians to prescribe the correct medications (Chaudhary, 2020). Nowadays, two drugs such as doxorubicin and cyclophosphamide have been prescribed in TNBC patients, which have shown promising results. Besides, other platinum drugs such as carboplatin and cisplatin are also being prescribed (Sikov et al., 2015). Furthermore, PARP inhibitors such as Olaparib, Velaparib, and PF-01367338, have been used as potential chemotherapeutics for TNBC (Ishino et al., 2018). Since the Wnt/b-Catenin, NOTCH, and Hedgehog signaling pathways have been reported in the development and progression of TNBC, the drug targeting of these pathways could be an important strategy (Aysola et al., 2013). To date, though, surgery, radiation therapy, and chemotherapy remain the mainstays of TNBC treatment, significant progress have been made in developing new therapeutic modalities, including targeted therapy, immune therapy, different gene editing CRISPR associated tools such as Cas9n (nikase), dCas9, CRISPR/Cas12, Prime editing and CRISPR/Cas9-directed gene therapy. Here, we have focused on various CRISPR associated gene editing tool, which has been utilized for TNBC therapy. Of these, CRISPR/Cas9 have been emphasized extensively.

## 2 Cas9n (Cas9 nickase)

Cas9n, alternatively referred to as Cas9 nickase, is a genetically engineered variant of the Cas9 protein originating from the CRISPR/Cas9 genome editing system (Gupta et al., 2019). The Cas9 protein contains two nuclease domains, RuvC and HNH, in its original form, and their primary function is to cut both strands of DNA. However, in Cas9n, one of the nuclease domains, HNH, is genetically mutated and rendered inactive. As a result, the HNH domain of Cas9n remains non-functional. Only the RuvC domain remains active, allowing Cas9n to cut or create a nick in single strands of DNA (Trevino and Zhang, 2014). Cas9n can reduce off-target effects compared to Cas9 with high efficiency, and the improves the cell repair machinery accurately. Cas9n can be used in various proficient tasks, such as creating breaks at specific locations in double-stranded DNA. For this purpose, two Cas9n molecules are combined and used together (Yee, 2016). Mixed-lineage kinase 3 (MLK3) is a mitogen-activated protein kinase that serves as a critical regulator in the process of metastasis in TNBC (Cronan et al., 2012).

MLK3 can activate various signalling pathways, leading to metastasis in TNBC. Such as the c-Jun N-terminal kinase (JNK) pathway that regulates cell motility and extracellular matrix degradation, while MLK3 activates the JNK pathway, thereby augmenting cell motility and conferring invasive properties (Rattanasinchai and Gallo, 2016). MLK3 also regulates EMT. For this purpose, it activates downstream transcription factors such as Snail, Slug, and Twist. These factors suppress the expression of epithelial markers and promote the expression of mesenchymal markers, resulting in the acquisition of a metastatic phenotype (Casalino et al., 2023). MLK3 has been observed to play a role in ECM remodeling and activation of proteases, such as matrix metalloproteinase (MMPs), which degrade the ECM. This facilitates tumor cell invasion and dissemination to distant sites (Katari et al., 2019). Therefore, previous studies have revealed that MLK3 plays a crucial role in TNBC. Rattanasinchai and Gallo used a TNBC model to investigate the role of MLK3 and found that it contributes to cancer through specific signalling pathways. They utilized CRISPR/Cas9n to edit MLK3 and as a result, observed a significant reduction in TNBC metastasis (Rattanasinchai and Gallo, 2016).

## 3 dCas9

dCas9, commonly referred to as deactivated Cas9, is a derivative form of Cas9 protein that has undergone modifications. Unlike the active Cas9, dCas9 lacks endonuclease activity, rendering it incapable of inducing double-strand breaks in DNA. Consequently, dCas9 can be employed for precise targeting of specific genomic regions without introducing any alterations or modifications to the DNA sequence (Wang et al., 2016)**.** In dCas9, both endonuclease proteins, RuvC and HNH, are rendered inactive by silencing key amino acid residues, they have. (Richter et al., 2016). Despite lacking DNA cleavage activity, dCas9 plays several important roles in genetic research and biotechnology. For instance, it enables the visualization of specific genomic regions, facilitates transcriptional regulation, and contributes to epigenetic modifications (Brocken et al., 2018).

The transcription factor ZEB1 (Zinc finger E-box-binding homeobox 1) plays a specific and crucial role in promoting epithelial-mesenchymal transition (EMT), a cellular process that occurs in embryonic development, tissue repair, and cancer progression. It involves the conversion of epithelial cells into mesenchymal cells, resulting to changes in cell morphology, motility, invasiveness, and more (Wu et al., 2020). ZEB1 inhibits several epithelial markers such as E-cadherin and occludin, which are responsible for maintaining cell-cell adhesion and epithelial cell polarity in TNBC (Moreno-Bueno et al., 2008). Furthermore, ZEB1 activates certain markers such as N-cadherin, vimentin, and fibronectin (Konradi et al., 2014), also regulating the genes involved in cytoskeleton remodelling, such as Rho GTPases and matrix metalloproteinase (MMPs) (Huang et al., 2022), Additionally, it influences various signalling pathways like transforming growth factor-beta (TGF-β), Wnt signalling, *etc.*, thereby contributing to functions like tumor invasion and metastasis in TNBC (Chen et al., 2016). Several studies and scientific evidence indicate that ZEB1 holds significant potential as a valuable agent for the identification and therapeutic intervention of TNBC. In a recent study, Waryah et al.employed a TNBC model and achieved complete silencing of ZEB1 utilizing dCas9. Consequently, they observed a remarkably high specificity and near-total suppression of ZEB1 under *in vivo* condition (Waryah et al., 2023).

## 4 CRISPR/Cas12

CRISPR/Cas12 is a gene editing tool, known as CRISPR/Cpf1. It is a derivative of the CRISPR/Cas system in which the term Cas12 refers to the CRISPR-associated protein 12 (Bharathkumar et al., 2022), and entirely similar to Cas9. The only difference being that it contains Cas12 protein. Cas12 has some distinct functions compared to Cas9, such as producing sticky ends during gene editing, whereas Cas9 produces blunt ends (Wang et al., 2021). This property of Cas12 contributes to its specific DNA manipulation techniques. It possesses remarkable versatility as a protein capable of precisely identifying and cutting target DNA, rendering it a potent tool with diverse applications, including gene editing (Pickar-Oliver and Gersbach, 2019).

Similar to other CRISPR variants, CRISPR/Cas12 is also capable of performing gene knockout or activation in TNBC, targeting genes that play a substantial role in its pathogenesis or therapeutic response (Yang and Zhang, 2023). To achieve this process, a guide RNA (gRNA) is designed, which directs Cas12 to the target gene. Once Cas12 binds to the target, it introduces double-strand breaks in the DNA and activates the DNA repair mechanism. During the repair process, incorrect nucleotides may be incorporated, resulting in mutations and decreased functionality of the gene (Zhang et al., 2021a). Furthermore, the utilization of a modified iteration of the Cas12 enzyme known as dCas12 have been also employed for gene activation. By combining it with transcriptional activators, specific genes can be induced, resulting in their activation. This technique holds great potential in facilitating the activation of tumor suppressor genes (Sultan et al., 2022).

## 5 Prime editing

Prime editing is an extremely remarkable genome editing technique that has been developed in recently. It has the ability to precisely modify the DNA of living organisms in a very accurate manner (Chen and Liu, 2023)**.** Prime editing is achieved through the integration of two primary constituents, namely, a modified CRISPR/Cas9 enzyme and a reverse transcriptase enzyme. The CRISPR/Cas9 enzyme acts to selectively target a precise genomic site, while the reverse transcriptase enzyme facilitates the meticulous modification of the DNA at targeted location (Hassan et al., 2021)**.** In the prime editing mechanism, the first step involves the creation of prime editing guide RNA (pegRNA) (Standage-Beier et al., 2021). It contains a targeted sequence that matches the desired editing site in the target DNA and an RNA template. The pegRNA is then introduced into the target cells along with a prime editing nuclease (PE2). PE2 is a fusion protein that consists of a Cas9 enzyme, a reverse transcriptase, and a prime editing linker (Martín-Alonso et al., 2021)**.** Inside the cell, the pegRNA and PE2 complex look for the specific DNA, they want to modify. The Cas9 enzyme cuts the DNA and creates a template made of a single strand (Choi et al., 2022). The reverse transcriptase uses this template to prepare the DNA for editing. While, copying the DNA, the edited instructions from the RNA template are included. Finally, the newly created DNA strand is used as a blueprint to fix the cut in the DNA, resulting in a changed DNA sequence that contains the desired modifications (Ochoa-Sanchez et al., 2021)**.** The prime editing mechanism has the potential to introduce a wide range of mutations in genes. It can target point mutations, insertions, deletions, and even gene replacements (Anzalone et al., 2019; Chen and Liu, 2023). Prime editing confers multiple advantages over previous genome editing techniques. These include enhanced precision, reduced off-target effects, and the ability to perform DNA editing without reliance on double-stranded DNA breaks (Anzalone et al., 2020).

## 6 Overview of CRISPR/Cas9 system

CRISPR/Cas9 technology, which was originally designed to protect bacteria from plasmid transfer and phage infection, has been repurposed as an effective RNA-guided DNA targeting tool for genome editing (Jiang and Doudna, 2017). Also, it has been reported that CRISPR/Cas9 system is found in 50% and 87% of the genome in bacteria and archaea, respectively (Ishino et al., 2018). CRISPR/Cas9 is a potential tool to delete, insert and rectify the sequence of any abnormal gene using *in vivo* and *in vitro* modes (Sabit et al., 2021). Furthermore, CRISPR/Cas9 has been demonstrated as a part of the adaptive immune system due to its specificity for target genes of interest (Chen and Zhang, 2018).

### 6.1 CRISPR/Cas9 editing mechanism

**FIGURE 1:**
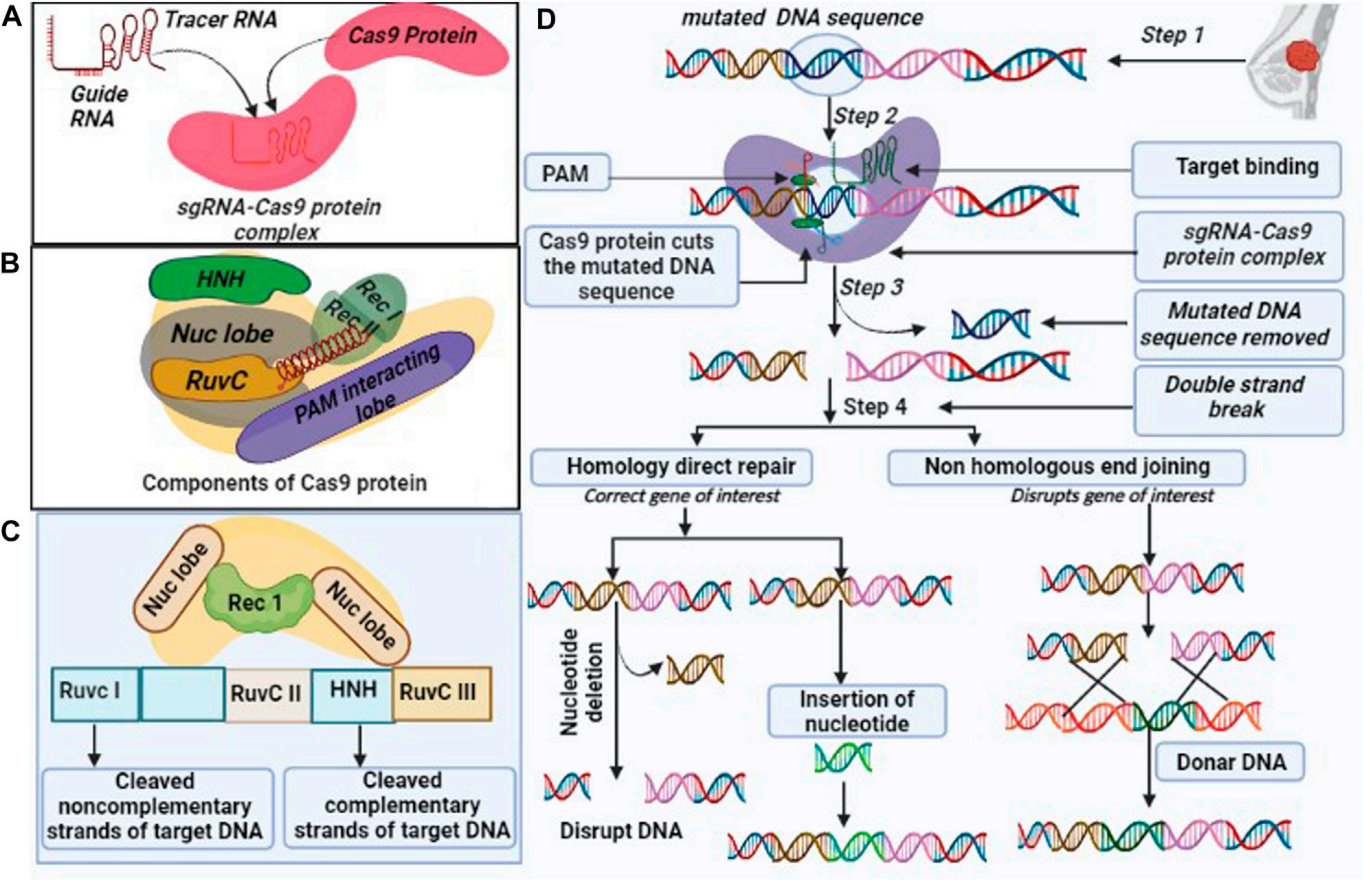
Overview of CRISPR/Cas9 **(A)**. Components of the CRISPR/Cas9 system: (i). Cas9 endonuclease which is responsible for cleavage of target DNA sequence, (ii) single guide (sg) RNA formed by the fusion of crRNA and tra-crRNA chimera. **(B)**. Cas9 includes multiple components such as Rec I, Rec II, NUC lobe (HNH and Ruv C are sub components) and a PAM interacting domain with their respective function, **(C)**. CRISPR/Cas9 protein complex cleaves the DNA sequence into non-complementary and complementary form, and **(D)**. CRISPR/Cas9 edit genome by following three steps: recognition, cleavage, and repair. The designed sg-RNA, guides Cas9 and recognizes desired sequence by crRNA, complementary base pair component. Cas9 recognized the PAM sequence at 5′-NGG-3′ and melt DNA by forming the DNA- RNA hybrid and activates for the cleavage. HNH domain of Cas9 cleaves the complementary strands and RuvC domain cleaves the non-complementary strands. CRISPR/Cas9 repair the dsDNA break by two pathways: Non-homologous end joining (NHEJ) and homology-directed repair (HDR). NHEJ repair dsDNA by an enzymatic process in the absence of exogenous homologous DNA, it is an error prone mechanism that can insert or delete the random DNA sequence. HDR is highly specific and requires homologous DNA templet.

CRISPR/Cas9 is made up of two components, i.e., Cas9 and single guide RNA (sgRNA). Cas9 is an endonuclease enzyme, which is composed of multi-components of protein. Also, Cas9 has unique structural and conformational properties (Pacesa et al., 2022), as it consists of two lobes, namely, the recognition (REC) and nuclease (NUC). REC lobe further divides into three regions REC1, REC2, and bridge helix (Cromwell et al., 2018). While NUC is made of three lobes, i. e., RuvC (RuvC I, RuvC II, RuvC III), HNH, and protospacer adjacent motif (PAM) interacting domain (Figure 1) (Song et al., 2016). sgRNA is made up of two components including CRISPR RNAs (crRNA) and trans-encoded small RNA (tracer RNA) (Figure 1). sgRNA join the Cas9 protein with the linker protein and form the active complex also known as the effector complex (Richter et al., 2012). crRNA is a 18–20 nucleotide base pair, that plays a crucial role in the identification of target DNA sequence. Further, crRNA is paired with the target DNA, and the tracrRNA act as scaffold for Cas9 nuclease to bind the target DNA (Manghwar et al., 2019). On the other hand, PAM sequence has 3 nucleotides, that confirms, specifies, and ensures the binding of the effector complex to DNA. Cas9 protein complex subunit RuvC and HNH have the catalytic activity (Richter et al., 2012; Asmamaw and Zawdie, 2021). The Cas9 subunit HNH nuclease domain cleaves the DNA strands that bind to crRNA. While RuvC nuclease domains cleave the other DNA strands and create the double-strand breaks (DSBs), after which two different DNA break repair mechanisms become active (Jiang and Doudna, 2017) (Figure 1).

### 6.2 CRISPR/Cas9 repair mechanism

CRISPR/Cas9-mediated repair pathways include non-homologous end joining (NHEJ) and homology-directed repair (HDR) mechanisms. NHEJ is an error-prone pathway, as it involves insertion or deletion, and does not require any template during repair mechanisms. It utilizes random nucleotides that produce default protein (Abbasi et al., 2021). The repair system has four complexes, such as KU complex, Cross-Complementing Protein type-4 (XRCC-4) complex, DNA end processing enzyme, and protein kinase DNA-PKcs (Abbasi et al., 2021). The KU complex protein has two subunits Ku 70 and Ku 80 and plays a significant role in the NHEJ mechanism because the repair mechanism is initiated with the binding of both subunits (Ku 70 and Ku 80) to blunt or near-blunt ends of the target DNA (Abbasi et al., 2021), which serves as a scaffold to recruit other NHEJ related factors to the damage site (Yang et al., 2020). The XRCC-4 and DNA ligase are comprised of 334 and 911 amino acids, respectively while XRCC4-DNA ligase IV complex stimulate the ligation of DNA ends (Chatterjee et al., 2015). DNA end-processing enzyme also called Polynucleotide Kinase 3^’^Posphate, is an end-process enzyme in the NHEJ repair, that potentially removes the 3^’^P group in DNA and phosphorylate the 5^’^-OH group during DSB repair. It also involves repairing single strands breaks (SSBs) using the SSB repair pathway (Chatterjee et al., 2015). Protein Kinase DNA-PKcs is a DNA-dependent protein kinase, made up of catalytic subunits of the PIKKs family (Phosphatidylinositol 3-kinase-related kinases), in addition to Ataxia Telangiectasia-Mutated (ATM) and ATM-, Rad3–related ATR that help in the double-strand breaks (DSBs) and single strands break (Yue et al., 2020; Peng et al., 2016).

HDR is a more accurate and suitable mode of repair mechanisms as the information is copied using the intact form of homologous DNA duplex, although it needs the presence of sister chromatids. This occurs in the S/G_2_ phase of mammalian cell cycles. HDR is mostly encountered in yeast species but NHEJ is crucial in mammals (Burma et al., 2006; Abbasi et al., 2021). The complete repair mechanism has been illustrated in Figure 1.

## 7 CRISPR/Cas9-based editing of oncogenes in TNBC

**FIGURE 2:**
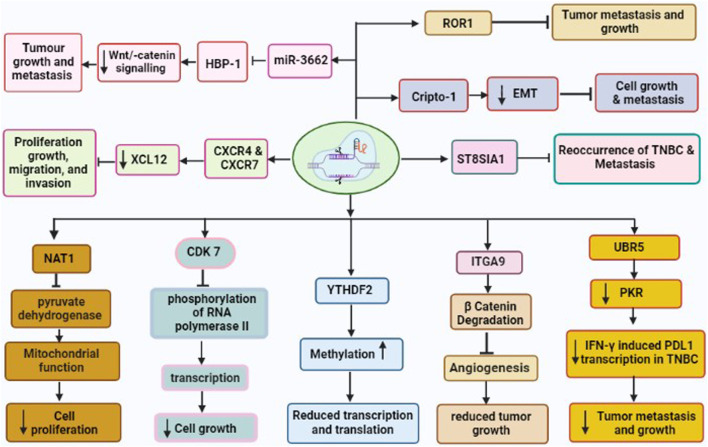
CRISPR/Cas9 driven gene editing of various TNBC oncogenes leading to reduced tumor growth and metastasis. These oncogenes are CDK7, NAT1, UBR5, YTHDF2, ITGA9, CXCR4 and CXCR7, Crypto1, ROR1, and ST8SIA involved in development and metastasis of TNBC.

Since, TNBC is caused by both genetic and epigenetic anomalies, using CRISPR/Cas9 to correct malignant genome/epigenome abnormalities could be a rational therapeutic approach (Chen et al., 2019). Also, some transcription factors that participate in cell-specific transcription regulation can show the distinctive characteristics of cancer cells, which suggests that transcriptional regulation might be an excellent approach to treating cancer (Drost et al., 2017). Utilizing these molecular features of tumors, such as genetic, epigenetic, and transcriptional defects to direct drug development may improve clinical outcomes, and lower the cost of screening. CRISPR, a potential tool for editing genes, can not only find and confirm genomic targets causing cancer, but also be used for editing, repressing, and epigenetically modifying cellular oncogenes in humans (Table 1) (Ahmed et al., 2021). There are currently several CRISPR screens done to find genes linked to tumor suppressors, oncogenes, and drug resistance. The editing of various TNBC oncogenes utilizing CRISPR/Cas9 has been shown in Figure 2.

**TABLE 1:** Latest research presented editing of various oncogenes using CRISPR/Cas9 for TNBC therapy.

Target gene	Cell line	CRISPR/Ca9 approach	Effects	References
ITGA9 gene	SUM159	knockout	CRISPR/Cas9 knockout romotes β-catenin degradation to suppress triple-negative breast cancer tumor growth and metastasis	Wang et al. (2019)
CXCR7 and CXCR4	MDA-MB-231	knockout	Decreased tumor cell proliferation, invasion, and tumor growth	Karn et al. (2022)
Cripto-1	JygMC(A)	knockout	inhibited tumor growth and pulmonary metastasis	Castro et al. (2015)
miR-3662	MDA-MD-231, BT-20, MDA-MD-157	knockout	Reduces the activation of Wnt/β-catenin signalling and stop the proliferation and migration of tumor cells	Yi et al. (2022)
UBR5	MCF-7 and MDA-MB-231	deletion	Reduce tumor growth and metastasis	Liao et al. (2017)
ROR1	MDA-MD-231	knockout	Suppression of metastasis and growth of TNBC	Pandey et al. (2019)
ST8SIA1	SUM159 and MDA-MB-231	knockout	Reduce tumor growth and metastasis by eliminating GD2+ BCSCs	Nguyen et al. (2018)
NAT1	MDA-MD-231	knockout	Affects the cellular metabolism, progression and metastasis Of tumor cells	Carlisle et al. (2020)
CDK7	BT549 and MDA-MB-468	gene editing	Increased apoptotic cell death and inhibit Tumorigenesis	Wang et al. (2015)
YTHDF2	MDA-MB-231	depletion	Proteotoxic cell death in tumor cells	Einstein et al. (2021)

### 7.1 CRISPR/Cas9 and knockdown of integrin α9 (ITGA9)

The migration, invasion, and epithelial-mesenchymal transition (EMT) of cancer cells have all been linked to ITGA9. Previous studies have revealed that ITGA9 is a key player in the Notch pathway and plays an intriguing role in the metastasis of rhabdomyosarcoma (Molist et al., 2020). In addition, ITGA9 has been found to have a strong association with patient outcomes across a variety of tumor types including breast cancer (Wang Z. et al., 2019). Furthermore, bioinformatics analysis on ITGA9 shows that its expression is significantly higher in TNBC than the other breast cancer sub-type. Increased level of ITGA9 has been linked to tumor metastasis and recurrence in TNBC patients. The use of CRISPR/Cas9 for the knockout of ITGA9, resulted in reduced cancer stem cell (CSC)-like properties, tumor angiogenesis, tumor growth, and metastasis by promoting β-catenin degradation in TNBC (Wang Z. et al., 2019).

### 7.2 CRISPR/Cas9 and Cripto1

Cripto-1 is a member of the TGF-β family, which is crucial for early embryogenesis, maintenance of stem cells, and metastasis of cancer (Ishii et al., 2021). It is also called Tdgf-1, an oncogenic GPI-anchored signaling protein, which participates in the regulation of generating the primitive streak, mesoderm, and endoderm layers, and establishing left/right asymmetry for developing body organs during the embryogenesis (Zhang et al., 2021b). Additionally, Cripto-1 has been demonstrated to involve inepithelial–mesenchymal transition (EMT) as a stem cell marker (Zhang et al., 2021b). The EMT is not only crucial for various processes such as embryonic development, fibrosis, and wound healing, but also in cancer invasion and metastasis. Furthermore, Cripto-1 has been also shown to interact with four Notch receptors, enhancing their post-translational maturation (Brandstadter and Maillard, 2019). It is well known that the Notch signaling pathway is involved in maintaining human breast cancer cells. It has been shown that CRISPR/Cas9-mediated Crypto-1 knockout inhibits cancer growth and metastasis. Therefore, Crypto-1 could be an important therapeutic target for TNBC (Castro et al., 2015).

### 7.3 CRISPR/Cas9 and knockdown of CXCR4 and CXCR7

XCL12 protein and its receptors CXC chemokine receptors (CXCR4 and CXCR7) play various roles such as cancer cell proliferation, growth, migration, and invasion (Wu et al., 2015). These chemoreceptors have been linked with TNBC development via several signaling pathways under both *in vivo* and *in vitro* models (Wu et al., 2015). Additionally, the upregulation of CXCR4 and CXCR7 has been associated with greater susceptibility to metastasis and poor prognosis of TNBC (Karn et al., 2022). Hence, the knockout of CXCR4 and CXCR7 could be effective target genes for the drug for the treatment of breast cancer including TNBC. A study by Yang et al. (2019) used the CRISPR/Cas9 to co-knockout both genes CXCR4 and CXCR7 and found significantly inhibitedrate of proliferation, growth, migration, and invasion of TNBC (Yang et al., 2019).

### 7.4 CRISPR/Cas9 and HMG-box transcription factor 1 (HBP-1)

The elevated level of microRNA-3662 (oncogene for TNBC) has been observed in breast cancer tissue (Yi et al., 2022). The miR-3662 knockout have been shown to inhibit both tumor growth and metastasis of breast cancer under both *in vivo* as well as *in vitro* condition (Agarwal and Gupta, 2021). The HBP-1 is a powerful inhibitor of Wnt/-catenin signaling and most likely the cause of miR-3662-mediated TNBC cell growth. Recently, Yi et al. have found that miR-3662-HBP1 axis regulates the Wnt/-catenin signaling pathway in TNBC cells (Yi et al., 2022). Due to its tumor-specific expression, miR-3662 could be a potential therapeutic target in TNBC. Hence, CRISPR/Cas9 mediated knockdown of miR-3662 could be an excellent approach in designing novel drugs in the TNBC therapy.

### 7.5 CRISPR/Cas9 and ubiquitin protein ligase E3 component N-recognin 5 (UBR5)

The UBR5 is a 300-kDa nuclear phosphoprotein that has been identified as a key regulator of tumor development, metastasis, and immune response in various cancers (Shearer et al., 2015; Fu et al., 2023). Reportedly, UBR5 is highly upregulated in TNBC samples, and stimulates the function of ERα induced proliferation via its ubiquitin ligase activity (Bolt et al., 2015). The whole-exon sequencing investigation of primary TNBC specimens also revealed an increased expression of UBR5, suggesting its role in the development of TNBC. Furthermore, CRISPR/Cas9-driven deletion of UBR5 has shown dramatic suppression of metastasis and growth of TNBC in an experimental murine model. In addition, the inclusion of UBR5 in the wild-type murine model, has reverted its complete function, whereas, in the inactive mutant strain, no such effect was observed (Liao et al., 2017). The lack of UBR5 has been associated to enhance the apoptosis, necrosis, and inhibition of tumor growth in TNBC due to its poor angiogenesis. The reduced tumor dissemination to distant organs has been observed due to the absence of UBR5, which induce abnormal EMT primarily through the downregulated expression of E-cadherin (Zhang and Weinberg, 2018). Recently, UBR5 has been shown as a very essential factor for IFN-γ induced PDL1 transcription in TNBC, due to the absence of E3 ubiquitination activity. RNA transcriptomic analysis has demonstrated that UBR5 may exert a systemic effect on genes involved in the IFN-γ pathway, where, it promotes the transactivation of PDL1 by enhancing the levels of protein kinase RNA-activated (PKR) and its signal transducers and activators of transcription 1 (STAT1) and interferon regulatory factor 1 (IRF1). Nevertheless, CRISPR/Cas9-mediated combined abrogation of UBR5 and PD-L1 expression offers synergistic therapeutic effects than each blockade alone, with a profound effect on the tumor microenvironment (Wu et al., 2022). Hence, CRISPR/Cas9 could be an important tool to mute the function of UBR5 and thus metastasis and tumor growth could be suppressed in TNBC.

### 7.6 CRISPR/Cas9 and receptor tyrosine kinase-like orphan receptor 1 (ROR1)

ROR1 is a type I transmembrane protein, that expresses during cancer and embryonic development and, has been identified as an oncofetal protein (Nicholas Borcherding, 2014). The aggressive behavior of various human cancers has been linked to the upregulation of ROR1. Promising outcomes have been seen under *in vivo* and *in vitro* studies involving therapeutic compounds targeting ROR1 (Chien et al., 2016). The elevated levels of ROR1 mRNA in breast tissue biopsies have been also associated with aggressive types of basal-like breast tumors (BL) and their migration to other parts of the body. Furthermore, ROR1 overexpression has been identified as prognostic marker for the development of TNBC (Chien et al., 2016). However, it would be an efficacious strategy to knockdown ROR1 through CRISPR/Cas9 to suppress growth and metastasis of TNBC.

### 7.7 CRISPR/Cas9 and ST8SIA1 genes

The sialylation process involves the addition of sialic acids into glycoconjugates, that is catalyzed by sialyltransferases (STs). ST8SIA1 is one of the STs family that has significant roles in the pathogenesis of different diseases such as lymphoblastic leukemia and colorectal cancer (Chang et al., 2018). RNA sequence analysis has demonstrated that ST8SIA1 is highly expressed in breast tissue of patients with TNBC and is positively associated with the mutation of tumor suppressor gene p53, which may lead to TNBC pathogenesis (Battula et al., 2017). Additionally, ST8SIA1 is involved in metastasis and recurrence of TNBC, implying its significant role in the development of TNBC. Utilizing CRIPSR/Cas9 to knockdown ST8SIA1, has been shown to block the growth and metastasis in the *in vitro* TNBC model (Battula et al., 2017). This offers the hint that CRISPR/Cas9 could be an important tool to inhibit the function of oncogene ST8SIA1 for TNBC therapy.

### 7.8 CRISPR/Cas9 and arylamine N-acetyltransferase 1 (NAT1)

The NAT1 is a metabolic enzyme, which catalyzes the phase-II xenobiotic compounds and expressed in almost every human tissue. NAT1 may also act on acetyl-Coenzyme A (acetyl-CoA) utilizing co-factor folate even when in the absence of its arylamine substrate (Stepp et al., 2015; Laurieri et al., 2014). It has been shown that NAT1 regulates the function of matrix metalloproteinase 9 (MMP9) in breast cancer cell models and protects from reactive oxygen species (ROS) during the shortage of glucose (Wang et al., 2018). The deletion of NAT1 has demonstrated the suppression of pyruvate dehydrogenase complex that leads to deregulated mitochondrial function (Wang L. et al., 2019). Additionally, other various reports suggested that the inhibition of NAT1 using small molecule and siRNA silencing, showed reduced invasiveness and proliferation of breast cancer cells (Stepp et al., 2018). Recently, the knockdown of NAT1 utilizing CRISPR/Cas9 in MDA-MB-231 breast cancer cell lines impacted the cellular metabolism depending upon its expression level. This study also shows that NAT-1 is crucial for the progression and metastasis of TNBC (Carlisle et al., 2020).

### 7.9 CRISPR/Cas9 and CDK7

The consistent transcription of oncogenes is regulated by super-enhancers, transcription factors, and co-factors (Hnisz et al., 2015). Additionally, the regulation of transcription requires a group of cyclin-dependent kinases (CDKs), such as CDK7, CDK8, CDK9, CDK12, and CDK13. Of these CDK7 involves in the phosphorylation of RNA polymerase II, which is important in the initiation and elongation of transcriptions of oncogenes during TNBC pathogenesis. In a seminal study, inhibited TNBC by the deletion of CDK7 through CRISPR/Cas9 suggested CDK7-dependent TNBC pathogenesis (Wang Y. et al., 2015).

### 7.10 CRISPR/Cas9 and YTHDF2

It has been shown that mutation in both MYC and RBPs (RNA Binding proteins) causes apoptosis, whereas single gene mutation either in MYC or RBPs, does not affect the growth of cancer cells (Einstein et al., 2021). Utilizing the CRISPR/Cas9-based library, more than 1,000 RBPs were screened in the human genome. Of them, 57 RBPs were found essential for the growth of cancer cells with highly upregulated MYC (Wheeler et al., 2020). Additionally, YTHDF2 is very important for maintaining the growth of TNBC cells, which reduced the methylated transcripts in high-level transcription and translation processes in the cancer cells where higher MYC expression was observed. Furthermore, YTHDF2 is not essential for the cancer cells, which are less dependent upon upregulated MYC for longer survival of TNBC patients, (Einstein et al., 2021), suggesting it could be a potential therapeutic target for the drugs to overcome TNBC.

## 8 CRISPR/Cas9 and identification of tumor suppressor genes in TNBC

Zinc-finger proteins (ZNFs) comprise approximately 1% of the total human genome. The studies suggested that ZNFs regulate cell proliferation in different cancers such as the liver, breast, and gut (Zhang W. et al., 2021; Li et al., 2017). CRISPR knockout-generated library was utilized to screen the different tumor suppressor genes (TSGs) in breast cancer cells (Shalem et al., 2014). Following this, a transcriptomic study on breast cancer cells with CRISPR/Cas9 deleted ZNF 319 identified ZNF319 as a tumor suppressor gene, that reduces the proliferation in breast cancer and therefore is involved in various potential signaling mechanisms and other biological functions (Wang L. et al., 2022).

Ferroptosis is a type of programmed cell death, that is dependent upon the presence of iron. It is known that the development of ferroptosis is influenced by the availability of lipid peroxides. Also, PKCβII has been reported to detect early lipid peroxides and increased lipid peroxidation has been associated with ferroptosis. The utilization of CRISPR/Cas9 mediated kinase inhibitor library screening has demonstrated that PKCβII participates in the lipid peroxidation process, an important for ferroptosis in MDA-MB-231 cells (Zhang et al., 2022). Hence, it suggested that knocking out of PKCβII by CRISPR/Cas9 may provide potential tumor suppressor gene targets for ferroptosis-associated disease treatment (Zhang et al., 2022).

## 9 CRISPR/Cas9 in overcoming drug resistance for TNBC treatment

Drug resistance is thought to be accountable for approximately 90% of deaths of cancer patients, and it is one of the major challenge in cancer therapy (Bukowski et al., 2020). Research findings indicate that an adequate number of genes linked to drug efflux, DNA repair, apoptosis, and various cellular signaling pathways have been associated with drug resistance (Haider et al., 2020). Of these, several genes have been targeted with CRISPR/Cas9 tool showing encouraging in terms of reduced drug resistance and enhanced efficacy of anticancer treatments (Vaghari-Tabari et al., 2022). Furthermore, the high-throughput CRISPR/Cas9 gene knockout screening library has been implicated to functionalize potential targets for modifying drug-resistance genes (Shalem et al., 2015). To identify the genes responsible for paclitaxel resistance, RNA sequencing combined with genome-wide sgRNA library screening has been utilized, and 8 candidates genes including histone deacetylase 9 (HDAC9) have been identified, which were associated with drug resistance in TNBC relapse patients (B et al., 2020). In another study, the increased expression of dual serine/threonine and tyrosine protein kinase (DSTYK) was observed in the survival of TNBC patients treated with anticancer drugs. Additionally, CRISPR/Cas9 mediated knockdown of DSTYK has dramatically increased the apoptosis of drug-resistant cancer cells in both *in vitro* (SUM102PT cells and MDA-MB-468 cells) as well as *in vivo* TNBC model (Ogbu et al., 2021).

Although TNBCs have abnormal activation of the MAPK pathway, the clinical outcomes of MEK-targeted therapy are very poor. CRISPR/Cas9 genomic library screening found that inhibiting PSMG2 (proteome assembly chaperone 2) enhances sensitization of BT549 and MB468 TNBC cells to the MEK inhibitor AZD6244. The knockdown of PSMG2 by CRISPR/Cas9 alters the normal function of proteasomes leading to the breakdown of autophagy-mediated PDPK1, which further improves AZD6244 (MEK inhibitor) and MG132 (proteasome inhibitor)-induced synergistic killing of tumor cell in TNBC mice model. Hence, proteasome and MAP kinase (MEK) inhibitors have been synergistically administered to reduce tumor cell proliferation (Wang X. et al., 2022).

CRISPR/Cas9 has been also utilized to screen the loss of function of genes that cause drug resistance in TNBC (Shu et al., 2020). Ge et al. explained the mechanism of drug resistance in cancer cells treated with JQ1, a BET bromodomain inhibitor (BBDI) in TNBC. Using CRISPR/Cas9, they found that the deletion of the rb1 gene caused resistance to JQ1 anticancer drug in TNBC. Hence, the function of rb1 is very important in TNBC to respond JQ1 drug. They also reported that paclitaxel is an inhibitor of CDK4/6 kinase/microtubule, and its combination with BBDIs such as JQ1 could provide a promising therapeutic response to TNBC resistance (Ge et al., 2020).

It has been reported that long noncoding RNA (lncRNA) transcriptional landscapes are responsible for the resistance to neoadjuvant therapy in TNBC. This study has shown the higher expression of five various transcripts of MALAT1 lncRNA in TNBC. Furthermore, CRISPR/Cas9 mediated deletion of MALAT1 sensitizes BT-549 TNBC cells to paclitaxel and doxorubicin, which indicates the potential role of MALAT1 drug resistance in TNBC (Shaath et al., 2021).

The mutation in BRCA1 (BRCA1m) is heterogeneous and therefore very hard to target. PARP1 (poly (ADP-ribose) polymerase) is a synthetic lethal partner of BRCA1, its targeting might enhance the chemosensitivity of drugs in TNBC. Utilizing CRISPR/Cas9, the deletion of PARP1 enhances the sensitization of anticancer drugs such as doxorubicin, gemcitabine, and docetaxel to mBRCA1 mutant TNBC cells, which suggest that PARP1 is also responsible for drug resistance in TNBC (Vaghari-Tabari et al., 2022). It is also well known that individuals are more likely to develop breast cancer in the presence of mutations in the tumor suppressor genes BRCA1 or BRCA2. Treatment for these patients required PARP inhibitors. Furthermore, it has been also demonstrated that knocking down of nucleotide salvage factor DNPH1 by CRISPR/Cas9 enhances the sensitivity of BRCA-deficient cells to PARP inhibitors by removing the toxic nucleotide 5-hydroxymethyl-deoxyuridine (hmdU) monophosphate (Fugger et al., 2021). Hence, CRISPR**/Cas9** along with inhibitors of PARP1 could be an important strategy for TNBC therapy.

Permeation-glycoprotein (P-gp) gene is a multi-drug resistant gene, which is mostly upregulated in approximately 41% of total TNBCs (Sun et al., 2020). The drug efflux due to P-gp has been identified as a key regulator for drug resistance in breast cancer. The P-gp inhibitors have shown the enhanced sensitization of anticancer drugs in breast cancers (Famta et al., 2021). Hence, CRISPR/Cas9 mediated deletion or silencing of P-gp along with P-gp inhibitors could be a very important approach in overcoming drug resistance in TNBC.

ATP-binding cassette transporter G2 (ABCG2) has been known to induce drug resistance in TNBC (Palasuberniam et al., 2015), Although, no report is available on the association between CRISPR/Cas9 and silencing of ABCG2, inactivation of tumor suppressor gene PTEN may enhance upregulation of ABCG2 (Palasuberniam et al., 2015), (Deepak Singh et al., 2021). Therefore, utilizing CRISP/Ca9 for deleting ABCG2 in combination with ABCG2 inhibitors to overcome the drug resistance in TNBC may be suitable therpeutic approach. CRISPR/Cas9 targeting all drug-resistant genes has been mentioned in Table 2.

**TABLE 2:** CRISPR/Cas9 target various drug resistance genes in TNBC to sensitize cells to anti-cancerous drugs.

Target gene	Cell line	Effects	References
MALAT1	BT-549 TNBC model	CRISPR/Cas9-mediated promoter deletion enhanced sensitivity of cells to paclitaxel and doxorubicin	Shaath et al. (2021)
PARP1	MDA-MB-231,MDA-MB-436	CRISPR/Cas9-mediated disruption sensitize cells to doxorubicin, gemcitabine and docetaxel	Mintz et al. (2020)
PSMG2	BT549 and MB468	CRISPR/Cas9-mediated knockdown Sensitized TNBC cells to drugs	Wang et al. (2022)
DNPH1	SUM149, DLD1	CRISPR/Cas9-mediated inhibition potentiates the sensitivity of BRCA-deficient cells to PARP inhibitors (PARPi)	Fugger et al. (2021)
(MRP2/ABCC2, P-gp/ABCB1, BCRP/ABCG2	TxR-HCC1806	CRISPR/Cas9-mediated knockdown enhance the uptake and reduce the efflux of anticancer agents	Kansara et al. (2020)
HDAC9, MITR/MEF2A/IL11	MDA-MB-231, MDA-MB-549, 231-PTX	CRISPR/Cas9-mediated suppression Sensitized paclitaxel resistance and provided a novel therapeutic strategy for TNBC patients to overcome poor chemotherapy responses	Lian et al. (2020)
P-gp	MCF-7 and MDA-MB-231	CRISPR/Cas9-mediated knockdown enhance sensitivity towards doxorubicin and rhodamine 123	Pote and Gacche (2023)

## 10 CRISPR/Cas9 library screening for identification of potential target in TNBC

CRISPR/Cas9 library is a potential tool to screen the mutations of genes involved in cancer pathogenesis (Chan et al., 2022). This screening method has involved four stages such as (a) library generation, (b) lentiviral transduction, (c) phenotypic screening, and (d) analysis of target genes. Even though TNBCs have abnormal activation of the MAPK pathway, and the clinical prognosis of MEK-targeted therapy for TNBC patients with a mutation of tumor suppressor genes such as PTEN, RB1, TP53 mutations, is very poor.

Further, most powerful and selective molecule, dehydrofalcarinol which is abundant in Desmanthodium guatemalense extract, was tested using a gene knockout screen by CRISPR/Cas9 to understand the mechanistic insight underlying the selective cytotoxic effect of dehydrofalcarinol against MDA-MB-231 cells, which represent the mesenchymal stem-like characteristics of TNBC subtype. This CRISPR/Cas9-based screening also found that gene *HSD17B11*, encoding 17β-hydroxysteroid dehydrogenase type 11, is highly expressed in MDA-MB-231 cells and responsible for the specific cytotoxicity of dehydrofalcarinol against MDA-MB-231 cells (Grant et al., 2020). Hence, it suggests that CRISPR/Cas9 genomic screening could be very potential in identifying the mechanism of potential natural compounds for their anticancer property.

Besides, cancer vulnerabilities in TNBC were studied using unbiased *in vivo* genome-wide CRISPR**/Cas9** screening, and the results showed a link between oncogenic and tumor suppressor pathways. It has been reported that key components of the mTOR and Hippo pathways are important in tumor regulation in TNBC. Additionally, it revealed that the pharmacological inhibition of mTORC1/2 and oncoprotein YAP efficiently suppresses pathogenicity in TNBC utilizing *in vitro* drug matrix synergy models and *in vivo* patient-derived xenografts. Also, Torin1-mediated inhibition of mTORC1/2 enhances macropinocytosis, whereas verteporfin-induced suppression of YAP results in the killing of cells in TNBC. Together, these results highlight the efficacy and reliability of *in vivo* CRISPR genome-wide screening for identifying novel and effective therapeutics for TNBC (Dai et al., 2021).

## 11 CRISPR/Cas9 and TNBC immunotherapy

**FIGURE 3:**
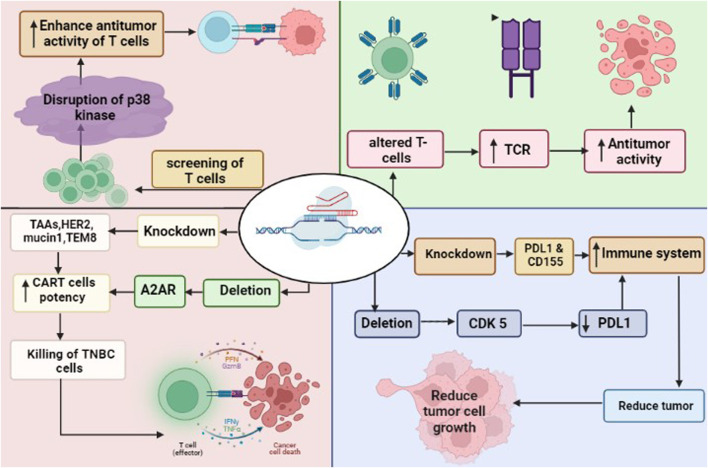
CRISPR/Cas9 in immunotherapy to attack TNBC cells. The deletion of CDK5 and knockdown of PDL1 and CD155 enhance the immune system. Similarly, the deletion of A2AR and knockdown of TAA such as HER2, mucin 1 and TEM8 enhance the potency of CAR-T cells leaded to killing of cancer cells. CRISPR/Cas9 driven screening of T cells found disruption of p38 kinase, that enhance the anti-tumor activity of T cells. The modified T cells by CRISPR/Cas9 increased the expression pf TCR (T-cell receptors), leaded to anti-tumor activity of T cells.

The dysregulation of the immune system is an important factor tumor development. Cancerous cells evade immune elimination through bypassing the defense mechanism, including interfering with immune cell activity and compromising the immune system in the tumor microenvironment. Therefore, a key approach to combat tumors may be in developing an improved immune system. Several issues related to immune dysfunction have been addressed from a variety of perspectives using CRISPR/Cas9-based genetic modification. By using the following pathways, CRISPR/Cas9 has been used to improve the anti-tumor immunity against breast cancer (Figure 3).

### 11.1 CRISPR/Cas9 targeting PD-1 and PDL1 interactions

The goal of immunotherapy is to stimulate the immune system to attack cancer cells. Overexpression of immunological checkpoint proteins, which ordinarily block autoimmune responses, may help cancers evade them (Topalian et al., 2015). These proteins prevent an immunological response by binding to receptors on the surface of immune cells. Several checkpoint proteins, such as CD155 and PD-L1, which targets the PD-1 receptor on immune cells, are expressed in breast cancers particularly TNBC (Li Y.-C. et al., 2020). Knocking down PD-L1 or its receptor with CRISPR/Cas9 may stimulate the immune system to attack the tumors of TNBC (Yahata et al., 2019). Additionally, downregulating PD-L1 expression through CRISPR/Cas9-mediated deletion of CDK5 has been proven to suppress tumor growth *in vitro* and *in vivo* (Deng et al., 2020). Growth inhibition in the *in vitro* and *in vivo* models of breast cancer suggests that shRNA-mediated reduction of CD155 may have therapeutic utility for breast cancer (Gao et al., 2018).

### 11.2 CRISPR/Cas9 and chimeric antigen receptor (CAR)-T cells

The CAR T-cells express CARs that recognize tumor-associated antigens (TAAs), and the knockdown of checkpoint proteins may be exploited to increase their activity (Li C. et al., 2020). There are different potential targets for CAR T-cell therapy in breast cancer, that include multiple TAAs, such as HER2, mucin1, and TEM8 (Bajgain et al., 2018). Evidence suggests that CAR T-cells targeting mesothelin, which is overexpressed in TNBC BT-459 cells, are more effective against cancer when PD-1 is knocked out using CRISPR/Cas9 (Hu et al., 2019). However, isolating T-cells from a patient and then editing them in an *ex vivo* setting is a time-consuming and arduous process. Although universal T-cells may reduce the requirement for isolation, the donor T-cells expressing class I human leucocyte antigens (HLA) and T-cell receptors (TCR) should be eliminated to stop graft-versus-host abnormalities (Ren et al., 2017a). Utilizing CRISPR/Cas9, HDR enables the simultaneous elimination of TCRs and HLAs along with the knockout of CAR-encoding genes. It has been shown that CRISPR/Cas9 can be used to introduce the anti-CD19 CAR into the TCR locus, resulting in efficient CAR production without exhausting T-cells (Dimitri et al., 2022). Multiplex techniques have been developed, which allow the simultaneous elimination of TCRs, beta-2 microglobulin (B2 M), a subunit of HLA-I, and other proteins like PD-1 and CTLA-4, leading to produce allogeneic CAR T-cells, which may have higher anti-cancer property against TNBC (Eyquem et al., 2017; Liu et al., 2017; Ren et al., 2017b; Dimitri et al., 2022).

The efficiency of CAR-T cells could be improved using CRISPR/Cas9. Adenosine is known to have immunosuppressive properties, which reduces anti-cancerous immunity via blocking T-cell function and activating adenosine A2A receptors (A2AR) (Vigano et al., 2019). The knocking down of A2AR utilizing CRISPR/Cas9 significantly increases the *in vivo* efficiency of CAR-T cells (Giuffrida et al., 2021). It is well known that T-cells have different phenotypic characteristics such as cell expansion, differentiation, oxidative stress, and genomic stress. CRISPR/Cas9-based screening of T-cells identified the disruption of 25 different T-cell receptor kinases. Among them, the deletion of p38 kinase has been shown to enhance the antitumor activity of T-cells, which suggests that p38 kinase is a key regulator in the regulation of CAR-T cells (Gurusamy et al., 2020).

### 11.3 CRISPR/Cas9 producing genetically modified T cell

T-cells could be modified genetically using CRISPR/Cas9 to produce highly expressed TCRs. The transfer of genetically modified T-cells in patients has shown enhanced anticancer activity than the endogenous T-Cells. Consequently, it is possible that endogenous TCRs compete with genetically altered TCRs in the patient, which might influence the potential of cancer immunotherapy. To overcome this, endogenous TCR-β was deleted using CRISPR/Cas9 in recipient cells and then the transfer of TCR-β T cells in cancer patients could exhibit better immunological response against cancer without competing with endogenous TCR (Fan et al., 2018). Thus, TCR in addition to CRISPR-modified T cells have shown a thousand times higher sensitivity to tumor antigen than normal TCR-transduced T cells. Furthermore, the γδ TCR + CRISPR generated altered T cells demonstrated higher expression of CD4^+^ and CD8^+^ T cells than standard TCR transfer in various leukemia (Legut et al., 2018). Hence, CRISPR/Cas9-generated modified T-Cells could be an efficient immunotherapeutic approach to overcome TNBC.

### 11.4 CRISPR/Cas9 targeting cellular adhesion molecules

Integrins are cellular adhesion molecules, that are present in the transmembrane of the cell and promote cells to bind to the extracellular matrix (ECM) (Hamidi and Ivaska, 2018). The dysregulation of integrins is associated with the development and migration of cancer via altering the ECM, leading to the survival of cancerous cells in the circulation (Hamidi and Ivaska, 2018). CRISPR/Cas9-mediated knockdown of integrin could slow down the tumor progression, metastasis, and colonization in TNBCs. Since knocking down of integrin a5 (ITGA5), has been reported to reduce cell migration and progression in other cancers such as lung (Ju et al., 2017), it indicates that integrin a5 may also be a key factor in TNBC pathogenesis.

## 12 CRISPR/Cas9 in generating *in vivo* model

Generating knockout mice using conventional embryonic stem (ES) cell methods is a time-taking, labor-intensive, and inefficient process. It takes many months to years to target ES cells through homologoue recombination, breeding of chimeric mice, then cross heterozygous mice to produce homozygous offspring. Complex crossings are necessary to produce mice with several genetic mutations. However, many issues are encountered while using mice produced by CRISPR/Cas9 eliminated ES cells or by microinjection of CRISPR/Cas9 components into the single-cell zygote. For instance, the introduction of changes frequently occurs at bi-allelic loci and is non gene-specific. Recently, it was reported that ES cells using the CRISPR/Cas9 technique may insert bi-allelic mutations up to five genes simultaneously (Nishizono et al., 2021). To do this, Cas9 mRNA and five gene-specific gRNAs were transfected into ES cells at the same time. This highlights the promise and effectiveness of this procedure, even though these mutations might spread when breeding the founder lines to create quintuple knockout mice. This study also reported a fascinating finding which revealed that ES cells would be no longer required to develop gene-modified mice. Instead, to delete particular gene products, Cas9 mRNA and gRNA were injected into one-cell stage embryos. As a result, knockout founder lines were produced, which in theory might be used to study the consequences of the deleted gene in mice (Qin et al., 2016). Hence, compared to conventional genetic engineering, CRISPR/Cas9 might create transgenic mice at a low expense for TNBC. A novel knock-in mouse model has been developed using CRISPR/Cas system, which can successfully introduce point mutations *in situ* in one or more endogenous genes in TNBC. According to this concept, the TNBC animal model could also be developed using CRISPR/Cas9 mediated deletion of BRCA1 and p53 that causes a lack of HR repair, genome instability, and a mutant phenotype (Annunziato et al., 2020).

## 13 CRISPR/Cas9 technology for diagnosis of TNBC

Currently, various techniques such as mammography, magnetic resonance Imaging (MRI), and ultrasonography are being used for diagnosis of TNBC. However, each of these techniques has a few limitations. Mammography is utilized for the diagnosis of localized tissues in the breast, and not for metastasis, where cancer cells migrated into different organs. Ultrasonography is not a very authentic tool for the diagnosis of TNBC (Chen and Lee-Felker, 2023). MRI exhibits higher sensitivity than ultrasound and mammography, but it has limited accuracy for diagnosis (Sha and Chen, 2022). Tissue biopsy is an invasive method for determining cancerous cell. However, in some cases, a biopsy might miss the cancerous tissues, if the needle beingdeflected from the precise region of interest. Also, it is highly expensive and causes trauma in patients. TNBC is a heterogeneous type of cancer, therefore a biopsy may not provide enough information about the type of cancer. Hence, a new diagnostic reliable method is much needed to identify the TNBC. CRISPR/Cas9 may serve as a highly sensitive and minimally invasive alternative for breast cancer diagnosis. For this, CRISPR/Cas9-based strategy could be employed to improve PCR method for TNBC diagnosis. First, non-specific DNA is deleted using Cas9 and cpf1 protein of CRISPR system, then these two proteins (Cas9 and cpf1) could recognizes PAM sequence before binding to target DNA (Deepak Singh et al., 2021). In this way, PCR could identify mutations involved in cancer development. Several studies have employed this CRISPR-based strategy to detect various mutations in different cancers (Safari et al., 2019). Hence, CRISPR-based PCR method could reduce the dependency on invasive techniques such biopsy-based immunohistochemistry for TNBC diagnosis.

It has been also demonstrated that alteration of genotypes occurs in response to hormone receptor modulation in TNBC (Chen and Russo, 2009). A CRISPR/Cas9-based PCR strategy using a chip for point-of-care diagnostics could be effective to trace these mutations (Hajian et al., 2019). It could also provide relevant information regarding specific mutations to healthcare professionals in TNBC patients, which could help in improving the course of their treatment. Although, limitations with this combined technique have been also seen. Therefore, extensive research work is needed before launching this CRISPR/Cas-PCR method in healthcare for the diagnosis of TNBC (Yang et al., 2019).

## 14 Integration of artificial intelligence (AI) with CRISPR/Cas9: an efficiency-enhancing approach

There are a variety of modulators that alter gRNA cleavage efficiency and cause off-target effects. These are PAMs, gRNA sequence, nucleotide used in gRNA, position-specific nucleotide composition, secondary structure, and epigenetic characteristics (Konstantakos et al., 2022). Given the importance of *in silico* gRNA design to improve CRISPR-Cas9-mediated gene editing, research is being focussed on maximizing gRNA design towards on-target efficiency and minimizing off-target effects (Chuai et al., 2017). A recent study has presented several algorithms that show promising results for increasing the activity and specificity of the CRISPR/Cas9 system by predicting the on-target and off-target effects of the CRISPR gRNAs (Henry et al., 2014). These forecasting methods have been crucial to expanding the utility of CRISPR and increasing its success rates. However, a subset of AI, i.e., machine learning (ML)-based algorithms can circumvent the fact that gRNA efficiency depends on interactions among parameters like cellular environment, experimental settings, gRNA, and target sequence. The on/off-target effects of investigating datasets can be predicted utilizing ML models trained with current datasets. There are currently three types of ML models based on (i) regression (Xu et al., 2015; Wilson et al., 2018), (ii) classification (Wilson et al., 2018; Chari et al., 2017), and (iii) ensembles (Peng et al., 2018; Hiranniramol et al., 2020).

The differences among these ML models depend upon the type of features utilized and the way the target site is presented (Konstantakos et al., 2022). High-precision target predictions for the CRISPR/Cas9 system are now possible due to the application of a subset of advanced machine learning, i.e., deep learning (DL) technologies such as artificial neural networks (ANNs). In the CRISPR/Cas9 system, DL models consist of many layers of computational nodes. The method accepts as input a matrix representation of a 23-nucleotide gRNA-DNA sequence. The input matrix is passed through a series of filers with varying sizes in the convolutional layer. To improve learning and prevent over-fitting, the following layer applies batch normalization to the output data from the previous layer. The third layer of pooling does additional filtering on the normalized data. The neurons in the many thick layers that receive the pooling layer output are all fully linked to one another. The last dense layer predicts whether the input is on-target or off-target and sends that information to the stop layer. In recent years, gRNA design and CRISPR applications have (Tsai et al., 2015) benefited greatly from the widespread use of ML and DL approaches. This approach helps to anticipate CRISPR gRNA activity and specificity scores (Chuai et al., 2017) by employing algorithms based on the ever-growing gene editing datasets reported worldwide. In addition, the ML and DL-based approaches are more productive and economical than experimental detection instruments like GUIDE-seq (Tsai et al., 2015), HTGTS (Frock et al., 2015), or IDLV (Wang X. et al., 2015).

Notably, the importance of ML and DL-based prediction techniques are not fully understood across a variety of cell types and species (Konstantakos et al., 2022). Therefore, research efforts have shifted to creating several species-specific tools, such as fryCRISPR for *Drosophila* (Listgarten et al., 2018) and CRISPR scan for zebrafish (Wilson et al., 2018), due to the high degree of variation between species. Algorithms have been described that help forecast the critical off-target consequences of the CRISPR/Cas9 system in addition to on-target efficacy. Some examples includstandard deep CNN (CNN_std) (Kleinstiver et al., 2016), Elevation (Lin and Wong, 2018), and Deep CRISPR (Wong et al., 2015). These findings have shown that avoiding unwanted off-target effects of CRISPR is possible with proper gRNA sequence design (Katti et al., 2022). Furthermore, truncating the gRNA, especially at the 5′end, has been demonstrated to lessen off-target effects (Wang G. et al., 2019). Therefore, the gRNA design algorithms represent a crucial step that could account for the effective implementation and expansion of the CRISPR/Cas9 system. However, before the gene editing system can be fully incorporated into treatments, the existing models and algorithms must be improved. These limitations include data imbalance and heterogeneity, a lack of training datasets, and inefficiency across species. The clinical and therapeutic uses of the CRISPR/Cas9 system need to have enhanced on-target activity with commensurate minimal off-target consequences.

## 15 Mode of delivery of CRSIPR/Cas9 *in vivo* and *in vitro* condition

As mentioned in the above sections, CRISPR/Cas9 has various roles in the deletion of abnormal genes and their repair. However, the implementation of this technique is not an easy task as it has to be delivered to the nuclei of target cells in different diseases. Therefore, the clinical outcome of CRISPR/Cas9 depends on its precise delivery to the target cells or organs (Wilbie et al., 2019; Wilbie et al., 2019). CRISPR/Cas9 delivery *in vivo* requires cargo and delivery vehicles. Cargo include DNA, RNA, and protein, which are delivered to the target cells. On the other hand, two modes of delivery for CRISPR/Cas9 include viral and non-viral (Lino et al., 2018).

### 15.1 Viral delivery systems

The ability of viruses to spontaneously transfer nucleic acids *in vivo* to various cell types makes them attractive candidates for the delivery of exogenous genes. Various viral vector types have been made available and modified to deliver gene editing tools (Raguram et al., 2022). Adeno-associated virus (AAV) and adenovirus are two of them that have been extensively utilized in preclinical research on Cas9-based genome editing techniques.

#### 15.1.1 AAV delivery system

The smallest and most basic animal virus AAV is a single-stranded DNA virus (Naso et al., 2017). AAV particles do not have an envelope and are made up of a SS-DNA genome of around 4.7 kb and an icosahedral protein capsid with a diameter of about 25 nm. The low immunogenicity, high efficiency, and good biocompatibility of AAV render it suitable in comparison to other viral delivery systems (Kaygisiz and Synatschke, 2020). Furthermore, recombinant AAVs rarely integrate into host genomes, unlike lentivirus and retrovirus. AAVs can also be directed to various target tissues *in vivo* by a variety of different capsid serotypes. These characteristics suggest AAVs as potential gene therapy vehicles; however, the low packaging capacity limits their use. Additionally, the AAVs could carry approximately 5 kb, which includes a transgene cascade and two flanking inverted terminal repeats (ITR). Hence, the space for exogenous transgene is limited up to 4.7 kb, which restricts it for delivery of CRISPR/Cas9 as the size of Cas9 protein is usually larger (Kaygisiz and Synatschke, 2020).

#### 15.1.2 Adenoviral mode of delivery

The adenovirus is an icosahedral, envelope-free virus of 90–100 nm in size with a large genome size of 36 kb, - (Lee et al., 2017). It has been reported as the most common viral vector, employed for the delivery of CRISPR, due to its larger packaging capabilities, genetic stability, higher transducing capacity and ease of production (Lee et al., 2017). Musunuru et al., utilized it for the delivery of cytosine base editors (CBE) in mice and found 28% enhanced editing efficiency of proprotein convertase subtilisin/kexin type 9 (Pcsk9), which lower the cholesterol levels in plasma in the murine model (Chadwick et al., 2017). Furthermore, Lieber et al. employed adenovirus to deliver adenosine base editor (ABE) in HSC *in vivo* to breakdown the blocker site of fetal hemoglobin promoter, leading to drastically upregulated fetal hemoglobin (Li C. et al., 2022). Although adenoviruses can introduce gene editing tools *in vivo* and create successful editing, their dosages are constrained during the application, presumably due to their high immunogenicity and cytotoxicity.

### 15.2 Non-viral mode of delivery system

Other delivery systems, like non-viral vectors, could be also employed to deliver exogenous genes via CRISPR/Cas9 in the *in vivo* animal models. Lipid nanoparticles (LNP) and natural extracellular vesicles from humans, are commonly utilized non-viral vehicles to deliver CRISPR for the targeted delivery. In the further section, we have explained both systems in detail.

#### 15.2.1 LNP delivery

Over the past few years, LNPs have received huge attention for the delivery of various gene editing technology (Finn et al., 2018), (Kenjo et al., 2021). LNPs have been extensively utilized as delivery vehicles for nucleic acids, such as siRNA and therapeutic microRNA. Typically, these genetic tools are in RNAs form, which is negatively charged and hydrophilic, that is repelled by similarly charged molecules in the plasma membrane of target cells, making their entry tough into the cells and high vulnerability to easy breakdown through ribonucleases (Kenjo et al., 2021). Thus, RNA must have some kind of protective covering so that it can be “transported” easily inside the cell by escaping degradative activities. As lipids are abundant in the cellular plasma membrane, the liposome coating can facilitate RNAs delivery into the cytoplasm through the membrane. To achieve this, liposomes need structural lipids and poly (ethylene glycol) (PEG)-linked lipids having positive charge and could bind to negatively charged RNAs (Paunovska et al., 2022). Recently, an efficient LNPs has been prepared utilizing novel amino-ionizable lipid, which was used in the delivery of Cas9 mRNA and sgRNAs and enabled 70% gene editing under *in vivo* condition of glioblastoma. This lead to inhibition of tumor growth by approximately 70% and improved survival by 30% (Rosenblum et al., 2020).

#### 15.2.2 Viral-like particle delivery system

VLP can also be used to transport gene-editing components in addition to LNP. They are a combination of non-infectious viral proteins, which encapsulate the target mRNA, protein, or RNP for delivery into the target cells or tissues (Lyu et al., 2020). Moreover, VLPs are produced from pre-existing viral backbones or viral capsid-like proteins, which share the same characteristics as their respective viruses, such as cargo encapsulation, endosomal escapement, and the capability to be reprogrammed to target multiple cell types (Lyu et al., 2020). VLPs, on the other hand, only provide gene editors temporarily in the form of mRNA or RNP, which decreases the likelihood of off-target gene editing and viral genome integration (Chandler et al., 2017).

Because retroviruses have several properties that are ideally suited for VLP (Zhang et al., 2015). These viruses constitute the basis for nearly all documented VLP architectures for delivering mRNA or protein cargo. Unlike non-enveloped icosahedral viruses, immature retroviral particles are round and often lack tight structural symmetry, allowing for more loading flexibility. In addition, larger retrovirus particles (100–200 nm) allow for a more physical area to package bulkier proteins like Cas9 (Zhang et al., 2015). Last but not least, retroviruses have a modular structure that allows them to target and infect a wide variety of cell types (Cronin et al., 2005). The capsid protein regulates packaging, while the envelope glycoprotein determines cell type specificity (Cronin et al., 2005). The ability to bind to different envelope glycoproteins shows that the VLP capsid structure, which efficiently encloses the target “cargo,” can be modified to alter its targeting specificity.

Lentivirus-derived VLPs have also been shown to carry Cas9 mRNA and sgRNA, which demonstrates its promising therapeutic potential of gene editing capacity (Yin et al., 2021; Ling et al., 2021). Additionally, engineered VLPs from mammal retrovirus protein PEG10 has also the capacity to deliver Cas9 mRNA and sgRNA, which shows rapid editing under *in vitro* conditions. Altogether, these pieces of evidence suggested that CRISPR/Cas9 could be delivered utilizing VLPs as vehicles.

#### 15.2.3 Extracellular vesicles (EVs) delivery system

The EVs are a diverse group of membrane vesicles secreted by many different cell types. Three distinct categories of EVs can be defined by their size and biological origins (Szatanek et al., 2017). (a) Exosomes are the smallest vesicles, with sizes ranging from 40 to 150 nm. They originate in the endocytic pathway, (b) Microvesicle (MV) are formed by outward budding of the plasma membrane and their size varies from 100 to 1,000 nm, (c) Apoptotic bodies, are synthesized during the apoptosis process and their size is more than 1,000 nm in diameter. All of these vesicles share similar characteristics of membrane-bound lipid bilayer as the plasma membrane (Hao et al., 2021), regardless of their mode of synthesis. Most cells continually secrete exosomes and microvesicles, which can be detected in the plasma, urine, breast milk, and saliva of a living organism. These molecules transport genetic instructions from 1 cell to other in the form of messenger RNA (mRNA) or microRNA (miRNA) (Colombo et al., 2014). The use of exosomes to transport bioactive compounds is an exciting new area of research. Its ability to transport intercellular nucleic acids and bioactive compounds across biological membranes imparts them a distinct edge as drug delivery vehicles. Electroporation (the most prevalent) as well as detergent-mediated permeabilization of membranes, freeze-thaw cycles, sonication, and extrusion have all been described as techniques for drug loading into exosomes (Haney et al., 2015).

Notwithstanding, it is still difficult to design synthetic carriers that can efficiently transport therapeutic materials like nucleic acids to their target regions *in vivo*. Efficient production of the vehicle and loading of the cargo, resistance to degradation, absence of immunogenicity, pinpoint targeting, adequate cellular uptake, absence or lower toxicity, discharge of the cargo into the appropriate subcellular compartment, and mediating the desired effects are all necessary (Duechler, 2013). The ability to fine-tune the characteristics of synthetic transfection carriers to suit these criteria is highly advantageous. Nevertheless, most of these traits are interdependent, and enhancing the unintended abnormality in another. In the case of RNA transfer, greater binding of the RNA molecule to the carrier could provide enhanced protection from a breakdown but may also reduce the release rate inside the cell. Enhancing the biodegradability to decrease toxic effects may reduce the serum half-life, while improving the processes for release into the appropriate cellular compartment may increase cytotoxicity.

Several aforementioned issues can be sidestepped by employing sEVs as a delivery system, as these have now been optimized as a competent transport vehicle. Nevertheless, important challenges with EVs include mass production, sufficient purification, and effective cargo loading. There are still substantial hurdles to be surpassed before the safe and effective clinical profile of sEV-based nanomedicines (Geng et al., 2021) could be established. The challenges in the mass production of EVs for medicinal uses include the requirement to standardize their isolation and purification to adhere to Good Manufacturing Practice (GMP) regulations. To ensure the safe implementation of EVs, modified regulations are necessary due to the complexity of their structures and the high degree of inter-vesicular variation. Also, there is much room for improvement in the targeting ability and loading efficiency of autonomous EVs.

He et al. used MVs secreted from epithelial cells to deliver CRISPR/Cas9 machinery to tumor cells (He et al., 2020). MVs from HEK293 cells were transfected with Cas9 loaded in plasmid encodes for sgRNA, which target the IQ-domain GTPase-activating proteins. This data suggested that the expression of GTPase-activating proteins is downregulated, which shows the MVs potential in the delivery of Cas9 against specific genes. Furthermore, HepG2 liver cancer cells were less viable after being exposed to the loaded MVs, and combinations with the multi-kinase inhibitor sorafenib showed synergistic effects. The *in vivo* effects of this treatment in a HepG2 xenograft mice model of the disease were quite impressive.

## 16 Limitations and challenges for CRISPR/Cas9

The CRISPR/Cas9 system has to be improved, as direct genetic repair of cancer gene mutations currently carries unmanageable risks. There are still many limitations and challenges associated with the therapeutic utilities of CRISPR/Cas9 which include off-target effects, lack of potential delivery system, and non-specific cleavage at the target. Off-target effects are related to any non-specific cleavage by CRISPR/Cas9 other than the target site. As CRISPR/Cas9 needs gRNA to direct Cas9 to cleave the specific target. Hence, gRNA may identify other sequences similar to the target sequence, that might lead to off-target cleavage, which may cause mutations and other risks such as genotoxicity associated with cancer progression. Genotoxicity is a big issue on the way to the editing mechanism of CRISPR/Cas9 (Tran et al., 2022; Periwal, 2017). Off-target effects are also a problem in the diagnostic potential of CRISPR technology for nucleic acid detection. Since they can result in either a false positive or a false negative, thus reducing the reliability of its clinical diagnosis. Although increasing the action of gRNAs to on-target activity while decreasing their off-target repercussions is a challenge for the CRISPR/Cas9 system (Periwal, 2017), several interesting new methods have been developed to detect and decrease the occurrence of these events. However, the off-target effects of the CRISPR/Cas9 editing tool could be prevented with use of various technologies Naeem et al., 2020). These include (i) the Use of ML and for the prediction of gRNA potential to guide Cas9 mRNA (Wienert et al., 2019); (ii) positive and negative control animal models for measuring off-target effects; (iii) employing high-fidelity Cas9 enzymes for increasing the on-target cleavage; (iv) numerous sgRNA design tools for achieving optimal sgRNA performance (Katti et al., 2022).

CRISPR/Cas9 has additional difficulties associated with their delivery inside target cells. CRISPR/Cas9 may also produce geno- and cellular toxicity if delivered via the two current methods such as viral and bacteriophage-derived vectors (Yang et al., 2016). To solve this issue, CRISPR/Cas9 system might be delivered effectively by being encapsulated in a lipopolymer with a cell-specific aptamer, which would allow for cancer-specific targeting and lower the toxicity in comparison to the usual viral and non-viral delivery methods (Liang et al., 2017). While the viral mode of delivery has a high success rate *in vivo*, there are major limitations, such as safety concerns Li et al., 2018). Researchers have also developed a wide variety of bio-based nano-vesicles such as extracellular vesicles to boost the therapeutic effect of breast cancer treatment in addition to the conventional delivery vehicles mentioned above (Sanna et al., 2014; Hu et al., 2018) Furthermore, to activate tumor suppressor genes using CRISPR *in vivo*, it has been demonstrated that a new nanoscale dendritic macromolecule agent could be delivered via intravenous injection (A. Kretzmann et al., 2019) Additionally, Li F et al. developed DNA nanostructures incorporating CRISPR/Cas9 and DNAzyme to co-deliver and sustain biological activity (Li F. et al., 2022). Upon triggering intracellular release, both Cas9/sgRNA and deoxyribonuclease are released, resulting in the simultaneous genetic regulation of tumor cells.

An important barrier to using CRISPR/Cas9 technology as a therapeutic tool is the absence of antigen-specific T-cells directed against the Cas9 protein. In a recent study, reported the immunological hazards in using CRISPR/Cas9-based clinical trials in humans (Chew, 2018). Another recent study demonstrated that human cells exhibited both innate and adaptive immune responses to Cas9 proteins from bacteria (Charlesworth et al., 2019). These findings cast serious doubt on the efficacy and, more importantly, the safety of the CRISPR/Cas9 approach to cancer treatment. To this end, extensive studies on Cas9-specific T-cells based immunotherapy are required. Cas9 that can elude the host immune system, or at least the fuse an immune-compromised molecule into the Cas9-harboring cassette, should be another area of focus for these studies (Sabit et al., 2021).

## 17 Conclusion and future prospects

CRISPR/Cas9 is an innovative technique that has been used successfully to cure a wide range of disorders, including cancer. It is cost-effective, highly specific and rapid, and does not require the use of multi-purpose mouse colonies. Owing to this, it gained a huge attention in the scientific community, particularly in the field of cancer biology. Concerns about the safety of using the CRISPR/Cas9 system for genetic enhancement and other applications have been raised in the social and ethical literature. Ethical concerns have been made over the potential for human germline genome editing, in which altered chromosomes would be passed on to future descendants via gametes, early embryonic cells, or fertilized eggs. However, there are a few issues that require fixing, like off-target effects and poor delivery systems. Scientists today utilize CRISPR/Cas9 mostly to turn-on tumor suppressor genes (TSGs) and turn-off oncogenes in mouse models. Additionally, ML and DL-based algorithms have significantly increased the CRISPR/Cas9 effectiveness about the diminished off-target effects, a crucial element in expanding its utilization in clinical therapies. The efficacy of the technique across species has been further improved by using species-specific CRISPR algorithms. It is important to remember that CRISPR could potentially address epigenetic modifications, another factor implicated in BC development along with genetic variations.

Despite some technical challenges in targeting oncogenes, the potential of gene therapy using CRISPR/Cas9 remains encouraging. In the future, customized therapy using CRISPR/Cas9-based techniques may prove to be an effective strategy for tackling the intricacies of diverse tumors and cancer treatment resistance. However, the success of CRISPR/Cas9-driven therapy will depend on well-designed sgRNA, careful monitoring for off-target effects, and efficient delivery. This method has shown great promise in treating chemotherapy drug resistance at every level of investigation.
